# How Was Nature Able to Discover Its Own Laws—Twice?

**DOI:** 10.3390/life11070679

**Published:** 2021-07-12

**Authors:** Addy Pross

**Affiliations:** Department of Chemistry, Ben Gurion University of the Negev, Be’er Sheva 8410501, Israel; pross@bgu.ac.il

**Keywords:** origin of life, dynamic kinetic stability, cognition, chemical evolution, systems chemistry

## Abstract

The central thesis of the modern scientific revolution is that nature is objective. Yet, somehow, out of that objective reality, projective systems emerged—cognitive and purposeful. More remarkably, through nature’s objective laws, chemical systems emerged and evolved to take advantage of those laws. Even more inexplicably, nature uncovered those laws twice—once unconsciously, once consciously. Accordingly, one could rephrase the origin of life question as follows: how was nature able to become self-aware and discover its own laws? What is the law of nature that enabled nature to discover its own laws? Addressing these challenging questions in physical-chemical terms may be possible through the newly emergent field of systems chemistry.

## 1. Introduction

The origin of life problem is striking in that it can be posed in several seemingly unrelated ways. Beyond the direct question: ‘how did life emerge?’, one could ask how it was possible for function and purpose—key life characteristics—to have emerged from an inanimate universe in which such characteristics would presumably have been non-existent? [1,2]. Functional biology, a central sub-discipline within biology [3], is rooted in life’s functional character, so that character must be inexorably linked to the origins question. Or, to pose the origin question in yet another way, how was an information processing system able to arise? Information transmission requires both a sender and a receiver—but how did senders and receivers come to be? How did matter become cognitive thereby enabling information transmission and processing to take place?

Yet another facet of the life problem remains no less perplexing. In exploring the nature of the universe, we must begin with certain axiomatic assumptions, primarily, the physical existence of the universe, together with laws that govern its behavior. That is the starting point from which all scientific endeavor commences. But a deep puzzle then arises. As will now be discussed, examination of living things reveals that through the life process, nature has acquired knowledge of its own laws, and developed a means of exploiting those laws. What then is the law of nature that enables nature to discover itself? Addressing that question, and how it relates to the origin of life problem, is the topic of this essay.

## 2. Discussion

### 2.1. Nature, the Technologist

We are all familiar with the concept of technology, the application of scientific knowledge for practical purposes. It is ubiquitous. Starting from simple applications, such as stone tools, human ingenuity has taken us far, and today’s range of technological applications is mesmerizingly large. Just think of modern communication, transport, health management, manufacturing. Michael Faraday and James Clerk Maxwell could not have imagined to what extent their pioneering work on the laws governing electromagnetism and electromagnetic radiation could change the world. However, while the idea that humankind has been able to exploit nature’s laws for practical purposes is obvious to all, there is a truly mysterious feature of technological development, one generally overlooked, that needs to be mentioned. Technological development and innovation did not start with us humans, but began literally billions of years earlier, before man ever set foot on the earth, in fact, at a time when all life on earth was still microbial. The striking reality is that nature’s technological achievements, even for simplest life, have far exceeded human technological achievement that we, in our anthropocentric arrogance, take so much credit for. A comparison of human versus natural technological capability is revealing.

Take flying for example. The Swiss mathematician, Daniel Bernoulli, uncovered the principles governing flight in 1738 when he discovered the relationship between pressure, density and velocity in a flowing fluid. That understanding eventually led to the Wright brothers taking their first flight almost two centuries later. Nature, however, has been exploiting those same aeronautical principles for millions of years, well before humans existed. Insects, for example, already took to the air some 320 million years ago, and flight would not have been possible without the insect’s ability to accommodate the specific structural and dynamic requirements that derive from those aeronautical principles. More remarkably, nature’s ‘understanding’ of aeronautical principles extends far beyond the ones laid down by Bernoulli. In bees, for example, the principles of flight are particularly complex and go beyond the thrust and lift concepts governing avian and aircraft flight. Careful study of how bees fly has revealed additional forces that come into being through wing rotation and vortex formation, as the short and stubby bee body would not support flight through just wing beating. The fluid dynamics enabling bee flight turn out to be different to those that enable a plane or bird to fly [4].

In the common house fly, the above mentioned technological prowess goes even further, demonstrating capabilities currently quite out of reach of human engineers. Flies have mastered the knack of routinely landing on a ceiling upside down, a remarkable aerodynamic feat. The successful inverted landing of a fly depends on a series of several perfectly timed and highly coordinated actions. First, the fly increases its speed, then it undertakes a rapid body rotation, much like a cartwheel, then it proceeds to extend its legs, before finally landing by a body swing that pivots around the legs that have attached to the ceiling, and all of this coordinated with the fly’s visual and other sensory capabilities. Such capability greatly exceeds current robot technology resulting in engineers utilizing high-speed videography of the fly’s inverted landing to learn how the fly carries out such a sophisticated maneuver [5]. Human engineers are not proud—they are more than happy to consult with common house flies to further their technological capabilities!

The aeronautical examples described above are just arbitrary ones from an almost infinite list of exceptional natural technological capabilities, and from many fields of human endeavor. For example, you might think that the chemical giant Dupont was the first to make polymeric materials, starting with nylon, the first synthetic fiber [6]. Nature, however, has been routinely manufacturing a range of natural polymeric materials for eons, whether silk, wool, or cotton. However, it is when one examines nature’s technological capabilities at the molecular level, that a truly astounding picture comes to light. No matter where one looks within the biological cell, one sees extraordinary technological capabilities, all quite staggering from a human technological perspective. Consider the capabilities of the ribosome, that microscopic entity located in the thousands in every living cell, and able to synthesize proteins from a supply of amino acids in an assembly-line type process, based on information coded into the cell’s DNA sequence. That molecular machine is able to churn out required proteins in the space of a few seconds [7]. The synthetic chemist, while able to combine amino acids to form peptides and simple proteins, can only gaze in wonder at the staggering efficiency and specificity of that ribosomal system.

Discussing the ribosomal protein generation system brings to the fore the issue of information processing technology. That leads directly to Claude Shannon’s landmark theory of information published in 1948 [8]. However, a moment’s thought indicates that nature has been applying information theory well before Shannon published his ideas and, together with Alan Turing and John von Neumann, initiated the information age. The role of nucleic acid sequences in governing life processes reveals that nature was into digital information transmission and storage billions of years ahead of those legendary human information pioneers.

My point is simple. Through an evolutionary process, nature has succeeded in uncovering and exploiting technological principles far beyond what we humans, even today, are able to achieve. Without doubt, nature is the ultimate technologist—the supreme information theorist, polymer scientist, energy engineer, electrical engineer, operations manager, architect, systems chemist, photochemist, synthetic chemist, molecular biologist, to name just a few of nature’s wide-ranging capabilities. It is staggering to realize that every living thing is continually exploiting nature’s laws, and able to do so far more creatively than any human technologist, past or present, and most remarkably, in the case of simple life, they are doing so without those life forms even knowing that such laws exist!

### 2.2. The Origin of Technology

The question now arises: how could such cognitive capabilities have come into being? How was nature able to become the ultimate technologist? Darwinian natural selection is clearly a significant part of the answer to that question. Darwinian selection is traditionally viewed as a blind algorithmic process, akin to what takes place during the execution of a computer program [9]. Such algorithmic processes have turned out to be enormously powerful, as clearly demonstrated by the exceptional technological innovations that computer programs themselves have brought into our lives.

However, pointing to the existence of such an algorithmic process is not sufficient. Something more is required. To better understand that additional requirement, consider a computer programmed to play chess. Thanks to the algorithmic process taking place within that computer, a computer may well outplay any human opponent. Interestingly, however, that chess-playing computer does not know that a human game called chess even exists, let alone that it is playing the game at any given moment. A computer only does what all computers do—it reduces any problem to a set of ‘dumb’ calculations. In similar fashion, the simplest living entity, a bacterium, is totally unaware of nature’s laws, but, again, through a step-by-step algorithmic process—in this case, that of natural selection—extraordinary technological advances came to be. Just as a chess computer does not know it is playing chess, a bacterium does not know that it is utilizing information theory to produce proteins, that it is actioning its immune system to protect itself from viral attack, or that it is propelling itself toward a food source.

However, the evolutionary process then takes a staggering, almost incomprehensible turn. Through that supposedly blind algorithmic process, an entirely new and implausible technological innovation emerged—mind. Nature appeared to have stumbled upon a new dimension of reality, one that seemingly extends beyond the physical. However, though that new dimension might appear separate from the physical, it is important to recognize that the non-physical dimension of mind can only be accessed through the physical dimension. Or, put differently, all indications are that the non-physical world is an evolutionary outcome rooted within that physical world, and therefore necessarily part of it.

The significance of that development is hard to overstate. It means that nature began to see, not just physically, as in sight, but conceptually, as in mind. Mind—the ultimate natural innovation, one we humans continually use, but have barely begun to understand. This remarkable innovation, in addition to its many practical benefits, enabled us humans to begin to discover natural law *consciously*, in stark contrast to the seemingly undirected algorithmic approach that nature followed for billions of years prior to that evolutionary development. The implications of that new pathway to scientific discovery cannot be overstated—*nature managed to uncover and exploit its own laws, not once, but twice!* Once, mindlessly—algorithmically; once, mindfully—consciously. Moreover, most wonderfully, the *mindful* way allowed us to begin to investigate, to understand, and to marvel at the *mindless* way. In the ultimate irony, through the technological innovation of mind, nature discovered a means of exploring itself. Through what started off as a seemingly blind algorithmic process, the cosmos became able to explore itself, to become self-aware.

The above discussion now enables us to rephrase the life question in an unconventional manner: how was a material system, capable of undergoing such an extraordinarily innovative algorithmic process, able to come about? Just as you cannot program a computer to play chess, if you do not have a computer on which that program can be installed, you cannot have technological advance through an algorithmic evolutionary process without there being a suitable material substrate—in this case, a simple life form—on which the blind algorithmic process can operate. How did that initial life form, the equivalent of the computer in the chess analogy, come to be? Hidden within natural law *there must exist the primal means by which nature was able to create such a system, one with the extraordinary potential to discover itself!* Agency, mind, technological innovation, scientific discovery—whether natural, whether human—all arose out of that primal system. However, it means that hidden away within the natural order, lie the physical means for exploring the natural order. It also suggests that the non-material aspects of reality—mind and consciousness—which accompanied the emergence of advanced life, can be physicalized, though the term is used here in a broader sense to the one usually employed. To support that less traditional view, I would argue that any manifestation of a physical system, such as mind and consciousness, even if it is not within traditional physical bounds, should be viewed as an extension of the physical world, and therefore subject to physical consideration. In any case, where natural selection was able to lead, human thought, like a good tracker, can surely follow. The path from mindless to mindful was presumably stepwise and rooted in material change, just like all evolutionary processes, and therefore, a legitimate subject for physical study, though one still to be explored. However, that is for the future. The current and primary challenge is more immediate: to discover how the first step along that extraordinary emergence process—from inanimate beginnings to simplest life—was at all possible. How could it have begun?

### 2.3. Kinetic and Thermodynamic Stabilities

Change characterizes the world, and the fundamental law that describes the direction of change in nature is the Second Law of Thermodynamics [10,11]. However, as we are all aware, that Second Law directive leads toward death, not life. The equilibrium state, the state that all physical-chemical systems are directed toward, is the antithesis of life. So how could the central principle governing natural change, one antithetical to life, lead to the emergence of life, to a system able to become cognitive and explore the universe?

One way of expressing the Second Law is to state that material systems are driven toward stability for thermodynamic reasons—to maximize the systems’ entropy [10,11]. However, through research in a relatively new area of chemistry termed systems chemistry [12], it has become increasingly clear that certain physical-chemical systems can be stable for *kinetic*, rather than thermodynamic reasons [13,14]. The persistent state that is achieved can be classified as one of *dynamic kinetic stability (DKS)* [15,16,17,18], as opposed to the more common stability kind, thermodynamic stability. Physical examples of dynamic kinetic stability are familiar—a water fountain or a waterfall. The dynamic, non-equilibrium steady state is maintained, as long as energy and material resources are continually provided.

However, the discovery by van Esch, Eelkema and co-workers in 2010 that a regular chemical system can be induced into such a persistent dynamic, energy-fuelled kinetic state [19,20] was a game changer. It made clear that there exists a new dimension of chemical possibility, a kinetic dimension. In that kinetic dimension, change derives primarily from kinetic factors, rather than thermodynamic ones, and revolves around the possible existence of thermodynamically unstable chemical systems maintained in a stable (persistent) dynamic kinetic state. What is particularly striking about this chemical state is that it can lead to unusual material characteristics, characteristics we normally associate with biological entities—self-healing, adaptive, even communicative [21]. Indeed, the DKS state depends on an energy-fuelled, irreversible, cyclical process, very much akin to life’s metabolic processes, which are also energy-fuelled, cyclical, and irreversible. The discovery of DKS systems is therefore able to contribute to the establishment of a more coherent physicochemical framework for accommodating living systems [22]. Given the evident generality of this new kind of chemistry, we have proposed a descriptive term for it, *dynamic kinetic chemistry* [22]. With the discovery of dynamic kinetic chemistry, life chemistry may have finally found its physical-chemical home!

A further striking feature associated with this newly discovered kinetic domain is that for any given chemical system in a particular thermodynamic state, there exist, potentially at least, an extensive array of structurally distinct energized kinetic states associated with that single thermodynamic state, and all accessible through the tuning of the system’s kinetic parameters [19,20,22]. In fact, biological systems have taken full advantage of the inherent multiplicity of kinetic states, as it is through the interplay of those multiple states that living cells are able to carry out key biological functions, for example, modulating the structural characteristics of the cell’s cytoskeleton, thereby enabling cell motility and material transport to take place [23]. Thus, the discovery of this new chemical domain appears to be a significant step toward the longstanding goal of better relating chemical and biological processes. Not surprisingly, great interest has been directed toward the preparation of such systems [21,24,25]. A detailed kinetic analysis of these systems has also been undertaken [26,27].

So where does replication come into the life picture? Just as a replicative capability in some chemical system on its own, is insufficient to explain the emergence of life, so a DKS state in isolation is also unable to explain the life phenomenon. However, when the DKS state is *combined* with a replicative capability, unexpected chemical possibilities become possible, in fact, the door to life’s emergence appears to open up. The kinetics associated with replication systems in the DKS state predicts that increasingly kinetically stable systems will form [28,29], and that realization helps lead to the formulation of a Persistence Principle [30], which can offer certain material insights beyond those provided by the Second Law. The principle may be stated as follows: *all material systems are driven toward more persistent forms.* As Grand pointed out with incontestable logic: “things that persist, persist, things that don’t, don’t” [31]. The point, however, is that persistence can come about for either kinetic or thermodynamic reasons, and that duality reveals the dual character of the stability concept: stability has *two* discrete facets: *time* and *energy*. Whereas the Second Law addresses stability primarily through the energy facet, the Persistence Principle does so through the time facet [30]. In fact, it is the kinetic power of replication that enables the Persistence Principle to extend its scope beyond that of the Second Law, as it helps explain the existence of highly persistent, yet thermodynamically unstable, systems.

The bottom line: the existence of replicative systems in a DKS state opens up an evolutionary path toward kinetically stable systems of enhanced persistence, where kinetic considerations, not energetic ones, play the dominant role. As a result, novel material characteristics can arise, eventually leading to life. Indeed, as will now be discussed, it would have been through the emergence of replicative DKS systems that would have led to the emergence of one of life’s most striking and central characteristics—cognition.

### 2.4. The Origin of Cognition

Cognition has been traditionally defined as “the mechanisms by which animals acquire, process, store, and act on information from the environment” [32], and is normally associated with neural organisms. More recently, however, cognition has been understood to manifest in aneural life forms [33,34]. In fact, it has recently been claimed that simplest life—bacteria—exhibits strikingly sophisticated cognitive capabilities, despite their aneural nature [33,34]. That realization leads to the obvious question: could a *chemical* system be able to exhibit rudimentary cognitive behavior, to perceive, process, and react to information? The answer: apparently, *yes*, and it is on this very point that the biological significance of the DKS state is further reinforced.

Recent work on chemical DKS systems suggests that such systems may have crossed the threshold for the emergence of a basic cognitive capability. Thus Merindol and Walther [21] have described how DKS systems are able to self-heal, sense, adapt, communicate—key cognitive characteristics—despite being unambiguously within the chemical domain. Moreover, theoretical considerations that have addressed the underlying basis for cognition, have concluded that the ontological essence of cognitive agents is that they are “dynamical systems” [35,36]. That view is satisfyingly consistent with DKS’s dynamic character.

However, with the chemical emergence of simple cognitive systems, a profoundly significant evolutionary event would have taken place. A cognizant chemical system that emerged, and which was structurally able to evolve and adapt to better exploit its environment in the drive toward increasing persistence, would find new structural and organizational possibilities. In fact, that process over extended time led to the emergence of the bacterial cell, a major milestone along the evolutionary path toward increasingly persistent forms. Incredibly, several billion years along, it led to the emergence of animals with neural systems and to the ultimate evolutionary discovery of mind. Significantly, however, the entire evolutionary process was governed by nature’s incessant drive toward increasingly persistent forms.

We are thus able to identify nature’s central principle that enabled nature to discover itself—*it is nature’s fundamental drive toward persistent forms.* In contrast to the common biological view that considers the evolutionary process to be a blind algorithmic process [9], there *is* a general directive for that evolutionary process. If, within a segment of physical matter, a replicative system is able to become activated into a persistent DKS state, then its natural drive toward increased persistence could lead that system to explore and exploit its world to further enhance its persistence, beginning with cognition, ultimately leading to consciousness, and all through an evolutionary process of continuing complexification. Indeed, it is nature’s inherent drive toward persistent forms that is responsible for life’s extraordinary complexity, and that tendency is already apparent at the chemical level. As noted by Otto and co-workers in their recent molecular Darwinian study, DKS stable replicators that are *more* complex, are selected over ones that are *less* complex, despite the intrinsically slower replication rate of those more complex ones [37].

Of course, once the energy source and/or essential replicative building blocks enabling the evolutionary process to continue, have been dissipated, cognition and the transient formation of information will also dissipate, just as all material information forms eventually dissipate. Ultimately, matter continues to barrel on toward its final destination of heat death.

What then was the precise chemical form of the system that led to the emergence of cognition, enabling nature to explore itself? We will likely never know, as the historic record has itself largely dissipated through the degrading effect of time. No matter, we are now in a position to characterize that chemical system in general terms. The chemical system that was potentially able to evolve into life, to become increasingly cognizant, to learn to explore the world outside of itself, was one that was *replicative, evolvable, and in the DKS state*, effectively the very same characteristics that define the living state. A physical-chemical description of what life is thus emerges. And that realization then leads us to the ultimate life challenge: can the human life form now proceed to synthesize unnatural life forms? Our technological experience suggests the answer is most likely *yes.* After all, much of human technological achievement has been to mimic nature’s creative achievements. It seems only reasonable to conclude that what nature was able to achieve mindlessly, we humans can achieve mindfully. The synthesis of simple chemical proto-life would now appear possible. However, this means that after centuries of scientific division, biology may be closer to becoming conceptually integrated within the physical sciences. We may finally be nearer to understanding life’s fundamental physical-chemical essence. The implications of such physical-chemical understanding would appear to be profound.

## Data Availability

Not applicable.
